# Perineural spread of recurrent cutaneous melanoma along cervical nerves into the spinal cord

**DOI:** 10.1259/bjrcr.20160122

**Published:** 2017-01-05

**Authors:** W. Phillip Law, Nolette Pereira, Kasturi Vaska

**Affiliations:** ^1^Qscan PET Centre, Brisbane - Gold Coast, Australia; ^2^School of Medicine, University of Queensland, Herston, Australia; ^3^Princess Alexandra Hospital, Brisbane, Australia

## Abstract

Perineural spread of malignant melanoma is rare but increasingly recognized as a potential mechanism of metastasis particularly in desmoplastic melanoma, which has neurotropic characteristics. In the head and neck, this form of melanoma spread affecting cranial nerves has been described; however, to date, only one case of melanoma spreading to the brachial plexus has ever been reported. We present a case of cutaneous melanoma recurrence below the right jaw with perineural spread along the C3 and C4 nerves into the spinal cord, something which has not been documented previously in the literature.

## Case report

A 45-year-old Caucasian male presented with right arm pain and a palpable mass below the right mandibular angle, 2 years after resection of a cutaneous melanoma in the right jaw. The initial resection 2 years prior was followed by a repeat local excision and level I–V lymph node dissection, with none of the 48 lymph nodes showing metastatic involvement. Ultrasound-guided biopsy of the recently developed mass confirmed recurrence of melanoma.

**Figure 1.**
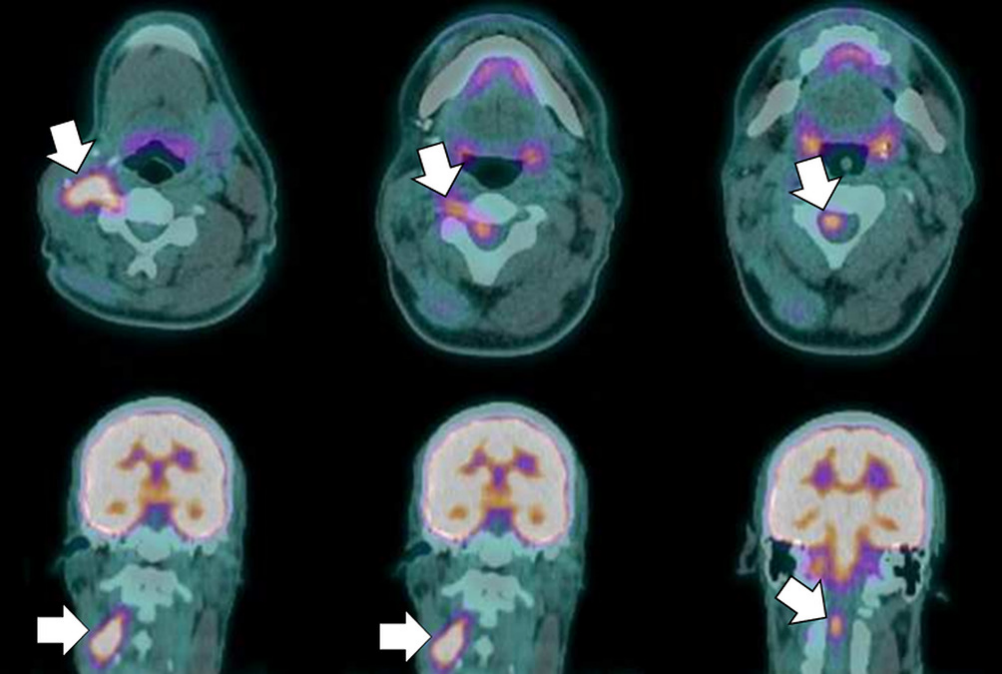
Positron emission tomography/CT axial and coronal images demonstrate an ^18^F-fludeoxyglucose-avid mass in the right side of the neck with tubular extensions along the C3 and C4 nerves into the spinal cord, indicating perineural spread (arrows).

^18^F-fludeoxyglucose (FDG) positron emission tomography (PET)/CT was performed for restaging of disease, demonstrating an intensely metabolically active tumour mass in the resection bed, with perineural spread along the right C3 and C4 nerves and contiguous invasion into the spinal cord (Figure 1).

**Figure 2.**
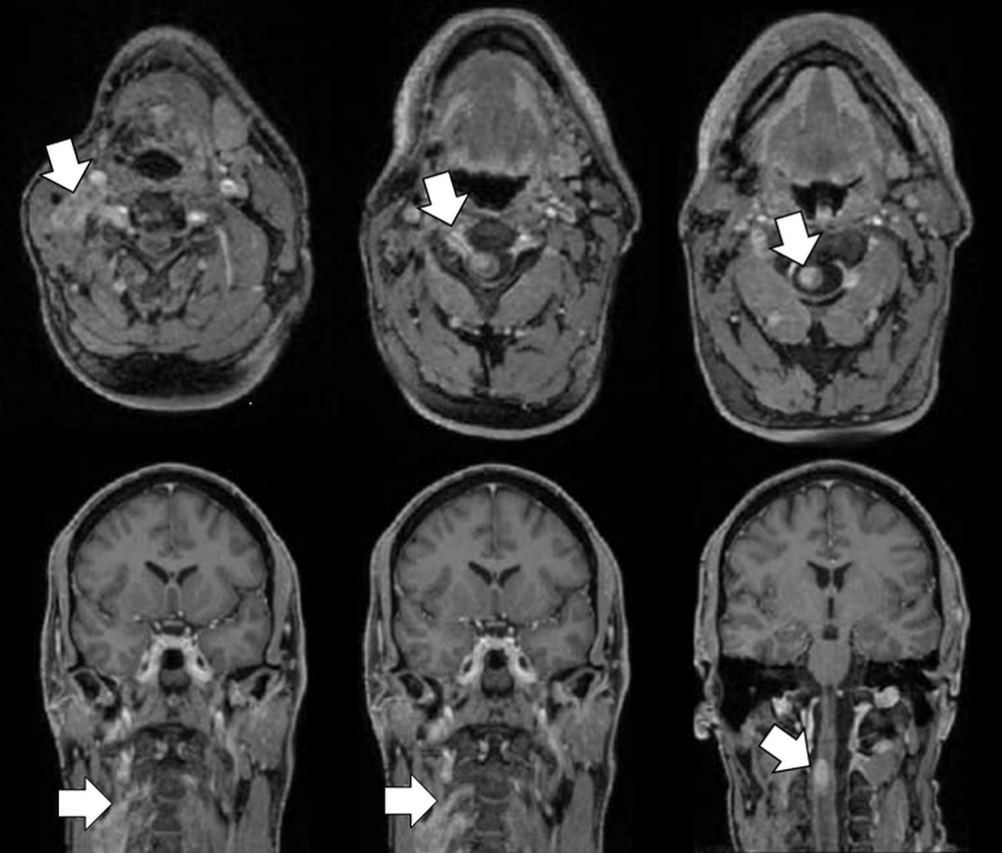
Post-gadolinium *T*_1_ fat-suppressed axial and coronal MR images demonstrate an enhancing mass in the right side of the neck representing local melanoma recurrence, with perineural spread along the right C3 and C4 nerves and contiguous invasion into the spinal cord (arrows).

MRI was subsequently performed for more detailed morphological characterization of local tumour recurrence and regional spread. This showed an irregularly enhancing soft tissue mass in the right side of the neck, with contiguous abnormal thickening and enhancement of the C3 and C4 nerve roots extending through enlarged intervertebral foramina into the right side of the spinal cord. Post- gadolinium *T*_1_ fat-suppressed images were particularly helpful in demonstrating the solid mass-like pattern of perineural nodular enhancement progressing retrogradely into the cervical cord substance (Figure 2).

Owing to the rapid growth of the recurrent mass in the submandibular region leading to ulceration of the overlying skin and significant discomfort, it was surgically resected for local control, and the patient was started on combined immunomodulatory treatment with ipilimumab and targeted radiation therapy.

**Figure 3.**
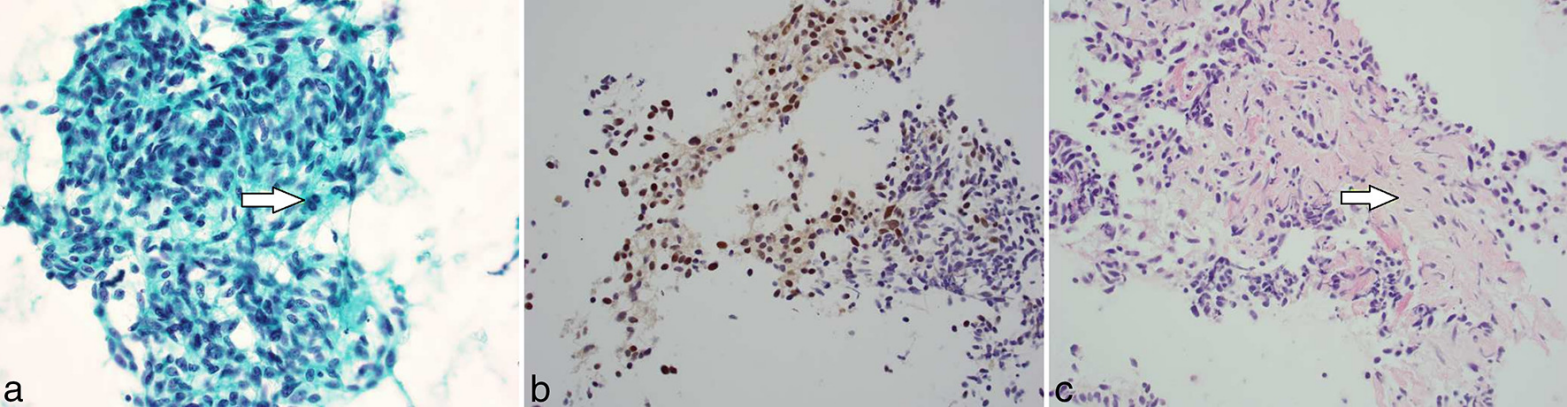
Photomicrographs. (a) Papanicolaou stain (original magnification x40)—fragments of tumour tissue containing spindle and epithelioid malignant cells with focal mitosis (arrow) morphologically consistent with melanoma. (b) Immunohistochemistry S100 (original magnification x40)—tumour cells exhibit nuclear and cytoplasmic staining. (c) Haematoxylin and eosin stain of cell block—desmoplastic stroma infiltrated by a single malignant cell (arrow).

Histopathologically, the resected mass contained desmoplastic stroma infiltrated by malignant cells with spindle and epithelioid morphology and enlarged nuclei containing prominent nucleoli. On immunohistochemical analysis, the cells were positive for S100 protein (Figure 3). The architecture of some nerves included in the resection specimen was disrupted and replaced by tumour.

## Discussion

Despite being the least common skin cancer—representing only about 5% of skin cancer diagnoses—melanoma is the most deadly, accounting for close to two-thirds of all skin cancer-related deaths. The incidence of melanoma worldwide has doubled over the past four decades, with the incidence in developed countries such as Australia, the UK and the USA having increased 10–15 times in this time. Approximately 20% of melanomas occur in the head and neck, as in this case.^1^

Perineural spread of the tumour is well documented in head and neck cancers, particularly squamous cell carcinoma, adenoid cystic carcinoma and lymphoma.^2^ It usually spreads along the facial nerve and branches of the trigeminal nerve; however, any cranial nerve can be involved.^2,3^ When occurring in skin malignancies, perineural spread is seen almost exclusively in squamous cell carcinoma. Perineural spread of malignant melanoma is rare^3^ but has been described affecting cranial nerves.^4,5^ Its spread along the brachial plexus has only been reported once;^6^ melanoma invasion along cervical nerves all the way back into the spinal cord has not been previously documented in the literature. The histological subtype that has a propensity for perineural spread is the desmoplastic variety.^5,7^ The affinity of desmoplastic melanoma for neural invasion appears to be related to a high expression of p75 neurotropic receptor involved in the migration of Schwann cells to the nerve.^8^ Regional recurrence is also common with this subtype of melanoma owing to its neurotropism (which can result in incomplete initial resection), although nodal metastasis is quite uncommon, and distant metastasis most frequently involves the lung, and less often the bone and the brain.^9,10^

MRI is the imaging modality of choice for morphologically depicting perineural spread of malignancy,^11^ although FDG PET has an increasing role in the detection of this pattern of tumour spread and for post radiotherapy follow-up.^12,13^ MRI provides excellent spatial depiction of tumours in the head and neck, while PET provides an excellent regional and whole-body screen of abnormal metabolic activity attributable to malignant recurrence and metastasis. Post-gadolinium *T*_1_ fat-suppressed sequences are particularly helpful for defining the extent of malignant spread through visualization of abnormal nerve thickening, nodular enhancement and enlargement of anatomical foramina and spaces, as well as denervation atrophy of the muscles. The addition of diffusion-weighted MRI may also address some of the disadvantages of conventional sequences in the imaging of the brachial plexus, namely the lack of signal contrast on *T*_1_ and *T*_2_ weighted sequences between the nerves of the brachial plexus and adjacent vascular structures, and the inability to produce three-dimensional images depicting and emphasizing the entire length of the nerves.

## Learning points

1. Perineural malignant spread can occur in the desmoplastic subtype of melanoma; when involving cranial nerves and spinal nerves this can result in direct intracranial or intraspinal invasion of tumour.
2. The incidence of melanoma has increased dramatically over the past few decades and radiologists should be familiar with the potential for this pattern of spread that can result in significant morbidity.
3. MRI and PET/CT are complementary to each other in the imaging evaluation of perineural spread in FDG-avid malignancies such as melanoma.

## Consent

Written informed consent was obtained from the patient for publication of this case report and accompanying images.
